# Assessing the feasibility, acceptability, and preliminary health behavior outcomes of a community-based virtual group health coaching for cancer survivors program

**DOI:** 10.1007/s00520-025-09295-y

**Published:** 2025-03-12

**Authors:** Nicole J. Berzins, Elizabeth Orsega-Smith, Michael Mackenzie, Mary Lou Galantino, Nicole S. Culos-Reed, Tara Leonard, Erika Narducci

**Affiliations:** 1 University of Delaware, The Tower at STAR, 3rd Floor, 100 Discovery Blvd, Newark, DE 19713 USA; 2 Stockton University, School of Health Sciences Office G-233, 101 Vera King Farris Drive, Galloway, NJ 08025 USA; 3 University of Witwatersrand Johannesburg, Johannesburg, South Africa; 4 University of Calgary, 2500 University Drive NW, Calgary, AB T2N 1N4 Canada; 5 Cancer Support Community Delaware, 4810 Lancaster Pike, Wilmington, DE 19807 USA; 6 University of Delaware, 11 Carpenter Sports Building, Newark, DE 19716 USA; 7 University of Delaware, The Tower at STAR, Room 228, 100 Discovery Blvd, Newark, DE 19713 USA

**Keywords:** Cancer, Group, Health coaching, Behavior change

## Abstract

**Purpose:**

The primary purpose was to assess the feasibility and acceptability of a group health coaching (GHC) program with cancer patients and survivors; secondarily, to determine the preliminary effects of GHC on several behavioral lifestyle factors.

**Methods:**

GHC was provided to people diagnosed with cancer via videoconference by trained health coaches across six GHC sessions over a 3-month period. Qualitative and quantitative data were collected. Data on recruitment, attrition, attendance, fidelity, retention, safety, and barriers and facilitators to implementation were assessed. Participant-reported outcomes collected via surveys included physical activity, eating habits, perceived stress, anxiety, depression, sleep, and quality of life, followed by post-program focus groups and in-depth interviews. Survey results were analyzed using repeated measures multilevel modeling. Qualitative data was analyzed using inductive thematic analysis.

**Results:**

Overall, 26 participants with a variety of cancer types attended an average of 74% of coaching sessions. The intervention was feasible to implement and found acceptable by participants and health coaches. Over the course of the intervention, there was a moderate increase in total weekly physical activity minutes (baseline = 365.25, follow-up = 510.30, *p* = 0.032, *d* = 0.50), and a small increase in weekly moderate-vigorous physical activity frequency (baseline = 4.07 bouts, follow-up = 5.44 bouts, *p* = 0.045, *d* = 0.39). Additionally, a moderate increase was found in functional well-being (baseline = 16.30, follow-up = 18.93, *p* < 0.001, *d* = 0.50).

**Conclusions and implications:**

GHC may be a feasible and acceptable way to promote behavior change for physical activity in cancer patients and survivors, reducing cancer burden and enhancing functional well-being.

## Background

### Cancer survivorship and a healthy lifestyle

There are currently more than 18 million people in the United States who have been previously diagnosed with cancer. Due to a growing aging population and advancements in treatments and screenings, the number of those living with and beyond cancer is expected to rise [1]. Cancer patients and survivors often experience additional health challenges throughout their lifetime such as long-standing effects of treatment, as well as depression and distress, which can affect physical health, mental health, and overall quality of life. Additionally, those with cancer are at an increased risk of cancer recurrence or developing a second primary cancer [2]. However, healthy behavioral lifestyle factors such as physical activity, diet, and sleep can help to reduce cancer burden by improving cancer outcomes, reducing symptoms, and improving overall quality of life [3–5]. It is therefore imperative attention be given to supporting health behavior change efforts with this population.

Despite knowing the benefits and having established guidelines for physical activity, diet, and sleep, less than a third of cancer patients and survivors discuss behavioral lifestyle factors with their physicians and these conversations take place less often than with their non-cancer counterparts [4–8]. Survivors are often motivated to make changes but struggle to get the information they need from their health care providers [6, 9]. While patients feel this information should be coming from their oncologist or primary care doctor, many physicians do not feel equipped to have these conversations nor do they feel well supported in how and where to refer patients for additional help when needed [10].

### Health & wellness coaching

To best meet the unmet needs of this population, a potential avenue outside of health care provider visits may need to be explored to support behavior change efforts. Health & Wellness Coaching (HWC) is an effective means of creating behavior change, particularly for improving perceived stress, physical activity levels, sleep, diet, and overall well-being in this population [11–13]. HWC utilizes an interpersonal relationship between client and coach whereby they work together to develop client-centered goals and use self-discovery in a non-judgmental environment, creating accountability towards fostering a health behavior change [14, 15].

While HWC has been shown to be effective, it can be costly and time consuming as coaches usually work with clients individually [16]. As such, HWC in a group format (i.e., GHC) has recently emerged. A scoping review revealed seven HWC interventions that have been conducted with cancer patients and survivors in a group format [17]. Studies appeared to be feasible and showed positive results for weight loss, diet, and quality of life, but results were mixed for physical activity, distress, and fatigue [17]. However, only three of the interventions utilized GHC as a standalone program, limiting our understanding of what GHC itself provides [18–20]. Of the three standalone interventions, the topical focus was either physical activity or a combination of physical activity, diet, and/or weight loss. No studies addressed the topics of stress management or sleep, despite growing evidence of their importance. These standalone studies were conducted entirely with females with gynecological [19], endometrial [20], or breast cancer [18] who were post-treatment at the time of the study, greatly limiting generalizability of the findings. Therefore, there is a need to explore how GHC may be utilized to encourage a variety of lifestyle behavioral changes with a wider variety of cancer types throughout the cancer continuum.

### Study purpose

Due to these limitations, a standalone virtual GHC intervention methodologically derived from prior one-on-one HWC research was designed [11]. This intervention had two aims: (1) To assess the feasibility and acceptability of a GHC program for cancer patients and survivors conducted through a videoconferencing platform; (2) to determine the preliminary effectiveness of the program on behavioral lifestyle factors including perceived stress, physical activity, sleep, diet, anxiety, depression, and well-being over a 3-month period.

## Methods

This feasibility study utilized a single group design to provide GHC to cancer patients and survivors. Due to COVID-19, the sessions were conducted using a secure videoconferencing platform, Zoom [21]. This project was a collaboration between a mid-Atlantic university and a community-based cancer agency that operates throughout the state. Both quantitative data in the form of fidelity checklists, attendance, and participant self-report surveys, and qualitative data in the form of post-program focus groups with participants and in-depth interviews with the health coaches were collected. Approval was obtained from the Institutional Review Board at the mid-Atlantic university (December 22, 2021/#1831200–1), and all participants provided written consent before participating.

### Recruitment

A convenience sample of participants were recruited using flyers, emails, and advertisements through the community-based cancer agency and senior centers throughout the state, through rehabilitation services at a local cancer center, and various avenues within the university. Those who showed interest were phone-screened to determine eligibility. Participants were considered eligible if they (1) were over the age of 18, (2) had been previously diagnosed with cancer (any type and stage), (3) were able to read and understand English, (4) were able to read and complete an online questionnaire, and (5) had the ability to access Zoom. There were no exclusion criteria independent of the inclusion criteria. Participants were verbally administered the Physical Activity Readiness Questionnaire (PAR-Q) to determine the safety or possible risks of exercising based on health history and risk factors [22]. If a person did not pass the PAR-Q, they were allowed to participate in the program but were asked to attain clearance from their primary care physician before goal setting around physical activity. If the eligibility criteria were met, informed consent was obtained.

### Program procedures

Participants met as a group for six sessions, every other week, over a 3-month period through Zoom. Sessions were led by health coaches trained through a National Board for Health and Wellness Coaching accredited training program [23, 24]. A trained research assistant also sat in on sessions to take attendance and record fidelity in real time. Sessions were also recorded using a videorecord feature in case anything needed to be reviewed. Cohorts were formed on a first come, first serve basis. The first session was approximately 90 min in length, with subsequent sessions approximately 45–60 min in length. Short educational modules lasting no more than 15 min were utilized at the beginning of each session to raise awareness around the topics of stress management, physical activity, sleep, and diet. A different topic was introduced each week and educational material was derived primarily from the American Cancer Society and National Comprehensive Cancer Network’s guidelines [4, 7, 25]. Material included a definition of the health topic, cancer-specific benefits, recommendations for cancer survivors, common challenges people face when trying to be healthier around that topic, and some suggestions for ways to make changes. Throughout the learning sessions there were built-in opportunities for participation, engagement, and discussions to ensure the educational component remained participant-centered, as well as priming participants for more in-depth discussions and goal setting later in the session. Following the educational material, health coaches facilitated in-depth discussions per participant’s interests, provided brainstorming activities, encouraged weekly goal setting and action planning, and helped participants address individual barriers to behavior change [14]. After each session, the health coach sent a follow-up email to each participant with an overview of the session including additional resources on that week’s topic. Overall emphasis was placed on ensuring modules were client-centered and client-directed per HWC philosophy [14].

### Data collection

All self-report surveys were collected in a REDCap database [26]. Each participant was sent an email with an individualized link to the surveys one to two weeks before the start of their cohort and following their last GHC session. Participants received a $25 gift card for each survey timepoint they completed. Qualitative data were collected in focus groups with participants after each cohort and in-depth interviews with the health coaches at the completion of the intervention. Both the focus groups and interviews were also conducted via Zoom. Each session was recorded via a video recording feature, then transcribed for thematic analysis.

#### Aim 1a: feasibility

Feasibility was assessed using the recruitment rate, attrition rate, attendance rate, fidelity, retention rate, safety, and barriers and facilitators to implementation [27]. Definitions for each metric as well as who collected it and when it was collected are described in Table 1. No priority was placed on any one metric.

**Table 1:** Feasibility metrics and definitions

Metric	Definition/tool used	Collected by	Time of collection
Recruitment rate	Number of people who consented out of the total number of current participants of the community-based cancer agency	PI	Post-intervention
Attrition rate	Number of participants who formally dropped out of the program out of the total number of consented participants	PI	Throughout the program
Attendance rate	Average number of GHC sessions attended by participants, tracked on an attendance sheet	RA	Each session
Fidelity	How closely the intervention was implemented as intended, assessed with pre-defined checklists developed by the PI	RA	Each session
Retention rate	Completion of follow-up measures	PI	Post-intervention
Safety	Number of adverse effects reported	PI	Post-intervention
Barriers and facilitators	In-depth interviews with the health coaches	PI	Post-intervention
	PI = primary investigator; RA = research assistant		

#### Aim 1b: acceptability and satisfaction

Acceptability was primarily assessed through post-intervention focus groups with participants and in-depth interviews with the health coaches, both conducted by the primary researcher. Satisfaction was additionally assessed at the follow-up timepoint using the Client Satisfaction Questionnaire-8 (α: 0.93) [28]. This Likert-scale survey has total scores ranging from 8 to 32, with higher numbers indicating higher levels of satisfaction with the program. It should be noted that this questionnaire was added after the start of the intervention, so only a portion of participants completed it (15/27 = 55%).

The Classroom Community Scale (CCS) was also collected at follow-up to assess group community and cohesion, specifically for an online learning environment. It has two Likert itemed subscales: one related to group connectedness and the other related to their feelings towards learning and whether their educational goals and expectations are being met. Each subscale can range from 0 to 40, with total scores ranging from 0 to 80. Higher scores indicate better cohesion (α: connectedness = 0.80; learning = 0.81; total = 0.90) [29].

#### Aim 2: behavioral outcomes

Behavioral outcomes were assessed through self-report surveys. The measures collected were as follows:

- Demographics and University Health Coaching Questionnaire

The demographic information collected at baseline included age, race, ethnicity, gender, marital status, and education. The University Health Coaching Questionnaire included medical information regarding the type and stage of cancer and their date of diagnosis.

- Perceived Stress Scale (PSS) [30]

The PSS is a 10-question Likert scaled survey used to determine participants’ psychological perception of stress within the last month. The higher the score, the more stress a person perceives with scores ranging from 0 to 13 indicative of low stress, 14 to 26 indicating moderate stress, and 27 to 40 indicating high perceived stress (baseline α: 0.90).

- International Physical Activity Questionnaire – Short Form (IPAQ) [31]

The IPAQ assesses physical activity across various intensities. It asks quantitatively the number of bouts of walking, moderate physical activity, and vigorous physical activity performed in an average 7-day period and the number of minutes spent during each bout. Additionally, the questionnaire assesses how many minutes per day are spent sitting.

- Pittsburgh Sleep Quality Index (PSQI) [32]

The PSQI assesses subjective sleep quality, sleep latency, sleep duration, sleep efficiency, sleep disturbance, and daytime dysfunction in adults. Total scores of 5 or more indicate poor sleep quality. While established clinical values or categorical cut-points have not been established for this scale, a systematic review found global PSQI scores of 8.31 for those in treatment, 7.10 for those post-treatment, and 7.33 at a follow-up timepoint (3 months – 1 year) [33] (baseline α: 0.82).

- Rapid Eating Assessment for Patients Short Form (REAP-S) [34]

This 13-item Likert scale measures eating habits. Total scores can range from 0 to 48, with higher scores indicating better eating habits. No clinical values or categorical cut-points have been established for this scale (baseline α: 0.70).

- Hospital Anxiety and Depression Scale (HADS) [35]

HADS is a Likert scored survey designed to detect the presence and severity of a relatively mild mood disorder likely to be found in non-psychiatric hospital outpatients. Scores for anxiety and depression are calculated separately. Scores for each range from 0 to 21 with scores of 0 to 7 categorized as “normal,” scores of 8 to 10 categorized as “borderline abnormal,” and scores of 11 to 21 categorized as “abnormal” (baseline α: anxiety = 0.78; depression = 0.76).

- Functional Assessment of Cancer Therapy: General, Version 4 (FACT-G) [36]

The FACT-G assesses general cancer-related quality of life (QoL) using Likert scales in four domains: physical, social/family, emotional, and functional. Scores are calculated for each individual subscale and a total score. Physical, social/family, and functional wellbeing scores range from 0 to 28, emotional wellbeing scores range from 0 to 24, and total scores range from 0 to 108 with higher scores indicating higher reported health related QoL. While clinical or categorical cut-point values have not been designated for this scale, one study found normative values for the cancer population to be: physical = 21.3; social = 22.1; emotional = 18.7; functional = 18.9; and total = 80.9 [37] (baseline α: physical = 0.80; social = 0.75; emotional = 0.83; functional = 0.86; total = 0.92).

## Data analysis

### Aim 1: feasibility and acceptability

For the quantitative feasibility metrics, count and descriptive statistics were conducted (proportions and frequencies). Mean and standard deviation was calculated for the Client Satisfaction Questionnaire-8 and the CCS.

For the qualitative data gathered from the focus groups and in-depth interviews, following transcription, the data were uploaded to the qualitative data analysis computer software Dedoose for inductive thematic analysis to identify common patterns and themes that emerged [38, 39]. With inductive thematic analysis, the themes are derived from the data itself [39]. The data was coded by the primary researcher who read the transcriptions in their entirety several times with the research questions in mind before beginning to generate codes. The researcher then began to identify key phrases from raw quotes and develop initial codes. Once initial codes were developed, the coder organized, merged, and refined the codes until broader themes emerged. The themes were then integrated with the subjectively reported experience of participants with the use of direct quotes to help substantiate themes and develop a fuller picture of the data [39].

### Aim 2: behavioral outcomes

Analyses were conducted on the baseline sample (*n* = 27) using IBM SPSS version 29 by the primary researcher [40]. Demographic information for the sample was characterized using frequency and percentages. To examine the overall effects of the program on facilitating change in each behavior, estimated marginal means models were computed for each instrument and corresponding sub-scales using repeated measures multilevel modeling [41]. In addition, Cohen’s *d*, a distribution-based effect size measure, was calculated for each outcome variable between baseline and program completion. Cohen’s *d* effect sizes can be interpreted as 0.20 as a small effect, 0.50 as a medium effect, and 0.80 as a large effect [42].

### Data integration

While not a mixed methods study, there are areas where the quantitative and qualitative data overlap. The qualitative data was analyzed first and themes found were compared to the quantitative findings when applicable to see where they might relate to one another. Similar to a convergent mixed methods design, this allows for areas of convergence or divergence of data to be compared and discussed to develop a more complete understanding of the concepts [43].

## Results

### Participant characteristics

The sample in this study was primarily female (92.6%), White (88.9%), non-Hispanic or Latino, (96.3%), married or partnered (54.5%), and well educated with a college degree or higher (70.3%). Participants were at various cancer stages and between the ages of 47 and 80. The primary cancer type was breast cancer (51.9%), with a wide variety of other cancer types also seen. Time since diagnosis (or most recent diagnosis if they had cancer more than once) at program start ranged from 3 months to 7.5 years, with the average time being just over 2 years (see Table 2).

**Table 2:** Participant characterization

Demographic	*n*	%	Cancer type	*n*	%	Cancer stage	n	%
Gender			Breast	14	51.9	Stage 0	4	14.8
Female	25	92.6	Pancreatic	2	7.4	Stage I	5	18.5
Male	2	7.4	Endometrial	2	7.4	Stage II	5	18.5
Participant age			Non-Hodgkin’s lymphoma	1	3.7	Stage III	8	29.6
45–54	6	22.2	Melanoma	1	3.7	Stage IV	3	11.1
55–64	9	33.3	Myxofibrosarcoma	1	3.7	Unknown or unspecified	2	7.4
65 +	12	44.4	Fallopian	1	3.7			
Race			Lung	1	3.7			
White	24	88.9	Prostate	1	3.7			
Black or African American	2	7.4	Pancreatic	1	3.7			
American Indian or Native Alaskan	1	3.7	Ampullary	1	3.7			
Ethnicity			Colon	1	3.7			
Non-Hispanic	26	96.3	Ovarian	1	3.7			
Hispanic	1	3.7						
Marital status								
Married	13	48.1						
Partnered/significant other	2	7.4						
Single	6	22.2						
Divorced/separated	4	14.8						
Widowed	2	7.4						
Educational attainment								
High school graduate	1	3.7						
1–3 years of College OR 2 yr College/Vocational/ Technical school graduate	7	25.9						
College/University Graduate	9	33.3						
Master’s Degree	8	29.6						
PhD or Equivalent	2	7.4						

### Aim 1a: feasibility

#### Recruitment

Participants were recruited between February and September of 2022. Forty-five people initially showed interest, 34 people were screened for inclusion, 27 signed the informed consent, and 26 participated in at least one GHC session. The community-based cancer agency sent an email about the intervention to all of their participants prior to the start of the program and again before recruitment ended. Emails were sent to 1,942 people for a recruitment rate of 1.4% (27/1942). However, this recruitment rate does not truly reflect those that may have opened the email or were interested (vs. all sent the email), and thus is a very conservative estimation of true recruitment.

#### Attrition

Four of the 27 consented participants dropped out from the program (14.8%). One participant dropped out after consenting but before starting the program, citing a change of mind. Additionally, three participants dropped out after attending at least one session for the following reasons: two participants decided GHC was not a good fit for them, and one had a non-cancer, non-intervention-related medical issue arise during the intervention.

#### Attendance

The overall attendance rate was 73.7%. Participants attended an average of 4.42 of the 6 sessions with more than half of the participants attending 5 or more sessions.

#### Fidelity

For this study, fidelity was defined as how closely the intervention was implemented as intended. Checklists were developed by the PI with components key to the program’s protocol listed to ensure health coaches were conducting the program consistently and reliably within and across cohorts [44]. The average fidelity across the entire program was 89.7%. Average fidelity for individual sessions ranged from 79.4% (Session 6) to 93.9% (Session 2).

#### Retention and safety

Twenty-three participants completed the follow-up survey measures for a retention rate of 85% of those who initially consented (23/27) and 100% of those who completed the program (23/23). For the focus groups, the retention rate was 48% of those who initially consented (13/27) and 57% of those who completed the program (13/23). No adverse effects from the program were reported.

#### Barriers and facilitators to implementation

During the interviews with the coaches, barriers and facilitators were explored. The first barrier was the use of Zoom. Second, one coach thought the health literacy of the protocol established materials might be at a higher level than the participants. However, having vetted materials created, including educational material during the sessions, follow-up email templates, session checklists, and the logistical support from the PI were seen as facilitators to implementation.

### Aim 1b: acceptability and satisfaction

Thematic analysis of the semi-structured focus groups with participants and interviews with the health coaches revealed six main themes: (1) overall impressions, (2) group benefits and cohesion, (3) perceived changes*, (4) program content, (5) program improvement, and (6) satisfaction. (*Theme noted by participants only.)

#### Overall impressions

Overall, participants reported positive experiences with the program. Participants appreciated the variety of topics covered and thought the program was a needed resource for cancer care as several participants stated they received very little information about these topics from their physicians. Several mentioned they felt prepared for treatment and treatment side effects, even potentially hospice or death, but they felt like no one prepared them for survivorship and therefore are interested in programs such as this.

Participant: What nobody prepared me for was doing well in surviving. It was kind of a, “Okay, you’ve finished treatment, you’ve had a clean PET scan, go live your life.” Well, how do I do that?

The overall impressions from the coaches were that they felt their experience was “valuable” and “rewarding.” They enjoyed being able to reach more than one person at a time and watching them learn from one another to be successful in their goals.

Coach: I found my experience in the program to be valuable… I enjoyed getting to know all of the participants that were members of my cohorts.

#### Group benefits and cohesion

The group experience was found to be extremely positive for most participants, with many appreciating being able to hear and learn from others while having the group for support and accountability. This was echoed by the coaches during their interviews as well.

Participant: I feel like the support we were able to give each other was as strong as the support we were able to get from [the coach].

Coach: Some people… were coming to this group out of fear of the unknown and they were really looking for stories and tips and tricks from the other participants who might have had a similar experience with treatments, with side effects, with how to deal with, you know, all of that… So, I think given the life-threatening nature of the work that we’re doing, we just have to give people more space to have those interactions.

The fact they were not a homogenous group was seen as a benefit.

Participant: Being part of this group really showed me how to give myself some grace because I really wasn’t where I thought I would be - new to my diagnosis, new to my treatment - and we started the group and having a wonderful bunch of ladies that were in different phases, different parts of their journey, um, different cancers share their experience and just show, um, no judgment but help and support was really beneficial to me.

This connectedness was also reflected in the CCS scores. The mean score for total classroom community was 57.81 (out of 80) or 72%. For the individual sub-scores, the mean connectedness score was 27.71 or 69% and the learning score was 29.91 or 75% (sub-scores are out of 40) (see Table 3).

**Table 3:** Comparisons of the effects of the intervention

Measures	Baseline	Follow-up	*p*-value	Cohen’s *D*	Confidence intervals	
	Mean (SE)	Mean (SE)			Lower	Upper
PSS	14.63 (1.25)	14.75 (1.29)	0.900	0.02	− 2.01	1.78
FACT-G						
Physical well-being	22.30 (0.72)	22.87 (0.77)	0.468	0.15	− 2.17	1.03
Social/family well-being	19.89 (0.97)	18.53 (1.02)	0.137	0.27	− 0.47	3.18
Emotional well-being	17.15 (0.85)	18.17 (0.88)	0.147	0.23	− 2.43	0.39
Functional well-being	16.30 (1.02)	18.93 (1.04)	< 0.001*	0.50	− 3.93	− 1.34
Total well-being	75.19 (3.06)	77.60 (3.15)	0.249	0.15	− 6.66	1.82
HADS						
Anxiety	6.93 (0.71)	7.11 (0.74)	0.746	0.05	− 1.37	1.00
Depression	4.48 (0.61)	4.12 (0.64)	0.475	0.11	− 0.67	1.39
REAP	32.52 (0.72)	32.51 (0.76)	0.99	0.00	− 1.43	1.45
IPAQ						
Weekly PA Frequency	9.07 (0.87)	10.57 (0.92)	0.086	0.33	− 3.21	0.23
Weekly MVPA Frequency	4.07 (0.67)	5.44 (0.70)	0.045*	0.39	− 2.69	− 0.03
Total PA Minutes per Week	365.25 (55.33)	510.30 (61.47)	0.032*	0.50	− 276.58	− 13.52
Total MVPA Minutes per Week	167.83 (33.13)	225.41 (36.67)	0.206	0.33	− 148.97	33.82
Weekly Sedentary Minutes	289.18 (33.93)	304.53 (35.99)	0.733	0.09	− 107.08	76.37
PSQI						
C1 Sleep Quality	1.48 (0.13)	1.25 (0.14)	0.142	0.34	− 0.09	0.56
C2 Sleep Latency	1.34 (0.18)	1.22 (0.18)	0.463	0.12	− 0.20	0.42
C3 Sleep Duration	1.52 (0.21)	1.22 (0.22)	0.189	0.28	− 0.16	0.77
C4 Sleep Efficiency	1.04 (0.21)	0.61 (0.22)	0.057	0.40	− 0.01	0.88
C5 Sleep Disturbance	1.44 (0.11)	1.53 (0.11)	0.509	0.15	− 0.33	0.17
C6 Use of Sleep Medication	1.07 (0.26)	0.92 (0.27)	0.489	0.12	− 0.30	0.61
C7 Daytime Dysfunction	0.85 (0.11)	0.80 (0.12)	0.731	0.08	− 0.23	0.33
Total	8.63 (0.81)	7.52 (0.85)	0.116	0.26	− 0.30	2.52
Classroom Community Scale						
Connectedness		27.71				
Learning		29.91				
Total		57.81				
Client Satisfaction Scale		26.07				

*PA *physical activity; *MVPA* moderate to vigorous physical activity

*Denotes significance

#### Perceived changes*

While a couple of participants felt they did not perceive any changes over the course of the program, most felt they saw improvements in one or more of the topic areas and developed better awareness of their actions and their potential impact on their health. Participants mentioned learning to not be as rigid and to give themselves grace when something did not go according to plan. Lastly, several participants mentioned no longer waiting until they get through certain treatment steps or events before taking steps towards a change. This was due to an understanding that they are capable of making a change now and that it can be beneficial, even while going through treatment.

Participant: I guess the biggest takeaway is that, um, you know, I’m not a victim of cancer, but I can be a warrior and I can be proactive and set goals.

Participant: Sort of my thinking was, after chemo, after radiation, after surgery, after, you know, oh cancer free, then I’ll do all these things. But I’m finding that as you’re going through, cancer is all the more reason that you need to have these activities during, you know, your treatment…..So, it kinda changed my, reset my mindset…and I find that you have to get up each day and make everyday count. So, that’s all. Just an overall change of my daily mindset. And so instead of putting it off, what can I do today?

#### Program components

While a few participants and one coach expressed interest in having sessions more often, most thought having sessions every other week was ideal as it allowed time to process the information they learned and gave them time to explore their goal(s) without feeling rushed. The sessions were considered well-structured by both participants and coaches. The topics of the educational components were found to be appropriate and valuable by participants, although as previously mentioned, one coach wondered if the health literacy level was too high for some of the materials.

Participant: I think the two weeks between was perfect because it gave you enough time to do your, do your tasks, to do your goals, you weren’t rushed and then gave you adequate things to talk about…I think, a week in between would have been too short and maybe only once a month or every three weeks, I think, I don’t want to say I would have lost interest, but it would have been maybe a little too long between sessions.

While some would have liked to have interacted with others in-person, many participants preferred the videoconference platform, citing perceived savings of gas and driving time, feeling it kept the sessions more task focused, and the accessibility of being able to connect despite limitations such as not being able to drive at night, being away on vacation, or not yet being comfortable with in-person gatherings post-COVID. Interestingly, while the coaches thought the Zoom sessions worked, given the choice they would have preferred to coach in-person, citing it might be more “meaningful” and “intimate.”

#### Program improvement

Longer sessions to allow for more in-depth discussion time were suggested, because hearing each other’s stories, struggles, and triumphs was voiced as being particularly valuable. This sentiment was also echoed by the health coaches, who sometimes felt “rushed to hit all of the deliverables on the checklist” while still maintaining authenticity.

Participant: Just creating a little more time for a little more opportunity to get in a little bit deeper, because when, when you bring together women with cancer, you know, and some of us at different stages… it can get pretty intense and we have things we want to talk about, share, especially because we’re with other women who understand. And it’s not easy to find that out in the general world, so I agree with having…a little more time for maybe to move into some deeper conversation.

Additionally, there was interest from some participants in expanding the nutrition and physical activity components, including adding some demonstrations or practical applications (i.e., chair yoga or cooking demonstrations). Several participants also thought a session on mindfulness would be a beneficial addition to the curriculum.

#### Satisfaction

The mean score for the Client Satisfaction Questionnaire was 3.2 out of 4, or 81% satisfaction. When asked, “How would you rate the quality of service you received?” 100% of participants selected “good” or “excellent” (3.53/4). When asked, “Have the services you received helped you deal more effectively with your problems?” 100% of participants selected “Yes, they helped somewhat” or “Yes, they helped a great deal” (3.33/4) (see Table 3).

Qualitatively, participants liked the program saying it was “helpful,” “great,” “a good experience,” and they “enjoyed everything.” The health coaches similarly said they found their experience to be “valuable” and “really enjoyed the format of coaching more than one person in a session.” When participants were asked if they would recommend the program to other cancer survivors, every participant said they would, although a couple of people qualified it by saying, “It’s got to be someone willing to open up and share with, you know, themselves and with the others in the group.”

### Aim 2: behavioral outcomes

Perceived stress levels, anxiety, and depression did not change significantly pre- to post-intervention. Participants reported a significant increase in total weekly physical activity minutes performed from baseline to follow-up (baseline = 365.25 min, follow-up = 510.30 min; *p* = 0.032, *d* = 0.50). A significant increase was also seen specifically in weekly moderate-vigorous physical activity frequency (baseline = 4.07 bouts, follow-up = 5.44 bouts; *p* = 0.045, *d* = 0.39). While total weekly physical activity frequency was trending towards significance (baseline = 9.07 bouts, follow-up = 10.57 bouts; *p* = 0.086, *d* = 0.33), no significant changes were found in weekly moderate-vigorous physical activity minutes or weekly sedentary minutes. The score for sleep efficiency trended towards significance (baseline = 8.63, follow-up = 7.52; *p* = 0.057, *d* = 0.40), but no significant changes were seen in sleep quality, sleep latency, sleep duration, sleep disturbance, use of sleep medication, or daytime dysfunction. No changes were seen in eating scores. A significant increase was found in functional well-being from baseline to follow-up (baseline = 16.30, follow-up = 18.93; *p* < 0.001, *d* = 0.50). Changes in physical, social, emotional, and total wellbeing, however, were not significant (see Table 3).

These data are somewhat juxtaposed with the qualitative data from the focus groups. While no significant changes were seen in perceived stress and anxiety in the quantitative data, several participants noted that goal setting within the program helped them to not feel as overwhelmed by their thoughts, giving them direction and something to look forward to. Furthermore, several participants mentioned having improved sleep, which was not seen in the quantitative data. Several people talked about having conversations with their physicians to obtain referrals to meet with a dietician, but not yet actively making changes to their diet which corroborates with the lack of change in the quantitative results. Many participants did mention being more active (biking, pickleball, walking, swimming, “moving more”), which provides additional validity to those quantitative findings as well.

## Discussion

This study assessed the feasibility and acceptability of a GHC program conducted through Zoom, as well as to assess the effects of the program on several behavioral lifestyle factors. Overall, this virtual program was deemed feasible to conduct due to the favorable retention, attrition, and fidelity rates, as well as the low risk to safety. However, it was not without its challenges. Due to the group nature of the program and the rolling start of the program, a cohort was unable to start until at least five people had committed to a particular program time. For two cohorts this took more than 2 months. Several people declined participation because of this delay in program start and one person who had consented dropped out. However, once participants were in the program, the attendance and attrition rates were found to be more favorable than other GHC interventions with this population, with rates for other programs to be 32% [45] and 53% [46] with attrition rates of 21% [46] and 42% [19], compared to 73.7% and 14.8% in this program. Online delivery likely enhanced attendance and lowered attrition by negating usual barriers such as transportation, scheduling, and distance to the study site [19, 20].

While the health coaches indicated Zoom and the health literacy level of the educational materials as potential barriers to implementing the program as intended, these barriers were not found in the participant’s qualitative data. Most participants noted feeling comfortable with the platform and thought the educational components to be appropriate for their needs. It is possible these challenges were not noted by the participants as the population was well educated, with 70% having a college degree or higher. Alternatively, prior research, especially with online courses, has shown higher rapport with the instructor or facilitator to correlate with higher perceived learning and greater success in the classroom [47, 48]. It is therefore possible the health coaches, having built rapport with participants, were able to teach the material in a way that was understandable by participants, despite the content of written material.

Additionally, the intervention was found to be highly acceptable, with coaches and participants reporting satisfaction with their experience at an overall rate of 81%. This supports evidence from prior cancer GHC interventions who found similar rates of satisfaction with their programs (8.7/10 and 83% satisfaction) [19, 20]. This is important because from a public health perspective, it is vital to note the need for additional resources to help bridge the gap between treatment and life beyond cancer in terms of behavioral lifestyle factors [6, 10]. Participants noted in the focus groups that they rarely receive, yet are often looking for, information on these topics. Due to the regimented nature of treatment, once it is over it is not uncommon for survivors to feel adrift and like they no longer have a plan, often making recovery feel harder than treatment [49]. Finding ways for community programs to connect with physicians and become a valued resource may be imperative to help support life beyond the cancer diagnosis [10].

Participants and health coaches provided useful feedback for improving the program as well. Their predominant suggestion was to lengthen the sessions to allow more time for discussions with others. Currently, there is a lack of consensus in the HWC community as to the optimal session length, with the majority of GHC sessions lasting 30 min or less [14, 17]. When considering individualized HWC, average sessions are often about 35 min per session [14]. Because in HWC the overarching goal is to help the client through the process of self-discovery and the development of individualized client centered goals, it is reasonable to assume more time may be needed for group sessions to allow everyone to feel heard compared to the time typically given to one individual [14]. Therefore, future research would benefit from a better understanding of the dosage effect and what amount of time may be best to elicit intended effects.

### Program benefits

Participants reported statistically significant improvements from baseline to follow-up in weekly moderate to vigorous physical activity frequency (a small effect size) and total physical activity minutes (moderate effect size). This is particularly interesting as participants were already meeting the national guidelines at baseline yet still reported increasing their physical activity over the course of the program. While prior research has shown some mixed results in this area, our results fall in the range of another cancer GHC intervention which showed an increase of 196 min per week [18] and a one-on-one cancer HWC intervention that saw an increase of 102 min per week [11]. While self-report and desirability bias should be considered, this potentially adds to the evidence that standalone GHC interventions may help to increase physical activity in this population without additional structured exercise components, if time and resources are limited [50, 51]. Additionally, improvements were seen in functional wellbeing from baseline to follow-up. Research has shown links between physical activity levels and functional QoL, with those participating in more aerobic activity minutes showing higher levels of functional QoL scores [52, 53]. Therefore, it is possible our population found an increase in functional well-being due to the increase seen in total physical activity minutes.

However, there were no significant changes in perceived stress, diet, sleep, anxiety, and depression, and most of the QoL subscales. This is somewhat surprising as three prior studies looking at HWC in a one-on-one format with the cancer population found improvements in physical activity levels (minutes and frequency), healthy eating scores, sleep quality and duration, anxiety and depression, perceived stress, and physical, functional, and emotional QoL [11, 13, 54]. There are several potential reasons why our results may have varied. First, our GHC program discussed a different health behavior topic each week. In one-on-one coaching, the topics discussed are guided by the client and it is possible to speak to all these topics within several visits, touch on them earlier in the coaching process, or address them more often across sessions due to the more individualized approach in one-on-one coaching, giving the participant more time to implement and reinforce strategies to change their behavior before the follow-up assessment [14]. In this study, participants were not introduced to the topics of sleep and diet until late in the program, giving less time to make improvements in these areas compared to physical activity. Participants commonly noted a change in their awareness of their current actions rather than changes to the actions themselves and more time may be needed to fully see these effects manifest as a change in behavior.

Additionally, the nutrition topic seems to be complicated for this population and more specific information might be needed for participants to sufficiently feel they are taking the right steps towards a healthier diet. Unless they have additional certifications, health coaches are not dieticians and have to be mindful of the scope of practice when discussing diet [14, 55]. While the education on this topic was guided by the American Cancer Society guidelines, several participants desired a more comprehensive overview of the topic due the overwhelming amount of information that can be found online. While our study was interested in understanding the sole effects of GHC there is evidence to support combining multiple facilitators (such as registered dieticians alongside a health coach) to see enhanced effects, and may need to be considered for future research [56].

Lastly, there is likely a ceiling effect for anxiety and depression, and to a lesser extent, perceived stress. When looking at the HADS cut points for “normal” vs. “borderline abnormal/abnormal”, our participants were classified as “normal” in both anxiety and depression at baseline, leaving very little room for improvement. Similarly, for perceived stress our population was barely over the “low stress” threshold at baseline.

### Limitations and strengths

While this study had several successes, the limitations of this study should be noted. The convenience sample and single group design were a limitation but was chosen due to the feasibility nature of the program. This design allowed the study to remain consistent with existing programming within the community partnership and to reduce participant burden. While common for this type of work, self-selection, self-report, and desirability bias still should be considered [50, 51]. Only slightly more than half of the participants attended the post-program focus groups. Due to this, it is possible some of the themes that emerged from the data were not representative of the group as a whole. Similarly, the Client Satisfaction Survey was added after the start of the program, so only a portion of participants completed it. However, information was gathered in all of the focus groups about program satisfaction to partially capture this metric from participants who finished before the survey was added. Lastly, due to the largely homogenous population and fairly small sample size, generalizability of the results is limited.

However, there were also some important strengths to note, including its inclusion of both qualitative and quantitative data. While quantitative data can give a snapshot of the effects, the addition of qualitative data from both participants and the health coaches provides an additional layer of richness and deeper understanding of the results that might otherwise be missed or misinterpreted. This data provides a more robust understanding of the feasibility and acceptability of not only those experiencing the program, but also those delivering it. Additional strengths are the high attendance rate, low attrition, and the use of fidelity checklists to ensure the intervention was being implemented as intended.

## Conclusion

With favorable attendance and retention rates, high fidelity, and the absence of adverse effects reported, this study provides evidence that GHC is not only safe, but well-tolerated by this population and feasible to implement. Provided in this format, individuals with a cancer diagnosis along the continuum of treatment and through survivorship are provided with peer support beyond what the coach alone can provide and allows for ongoing learning and connection with others, despite participants having different cancers and being in different places in their journey. Additionally, findings from this study provide evidence that GHC programs may be able to improve physical activity levels and physical functioning, although more research is needed to determine how these improvements compare with a structured exercise intervention with this specific population. While successfully delivered, valuable insight was provided by participants and coaches to help optimize future work with this population. Future research should include randomized clinical trials, powered accordingly and quantitative metrics to capture dosage of physical exercise. As GHC appears to be acceptable to those living with and beyond cancer treatment and it is feasible to implement, lessons from this study can help inform future work in the field.

## Data Availability

The datasets generated during and/or analyzed during the current study are available from the corresponding author on reasonable request.
